# How do enhanced videos support generative learning and conceptual understanding in individuals and groups?

**DOI:** 10.1007/s11423-023-10275-4

**Published:** 2023-08-25

**Authors:** Alessia Ruf, Carmen Zahn, Anna-Lena Roos, Klaus Opwis

**Affiliations:** 1 School of Applied Psychology, University of Applied Sciences and Arts Northwestern Switzerland, Olten, Switzerland; 2 Faculty of Psychology, University of Basel, Basel, Switzerland

**Keywords:** Video-based learning, Enhanced tools, Collaborative learning, Hypervideos

## Abstract

Videos are an increasingly popular medium for supporting learning in various educational settings. Nowadays, newly designed video-based environments contain enhanced tools that allow for specific interactions with video materials (such as adding annotations and hyperlinks) which may well support generative learning and conceptual understanding. However, to exploit the potentials of such enhanced tools, we need to gain a deeper understanding on the learning processes and outcomes that go along with using these tools. Thus, we conducted a controlled laboratory experiment with 209 participants who were engaged in learning a complex topic by using different enhanced video tools (annotations vs. hyperlinks vs. control group) in different social learning settings (individual vs. collaborative learning in dyads). Findings revealed that participants who learned with hyperlinks and participants in collaborative settings created hypervideo products of higher quality than learners in other conditions. Participants who learned with annotations assessed their knowledge gain higher and had higher results in conceptual understanding when they experienced low cognitive load. With our study we contribute new original work to advance cognitive research on learning with enhanced video learning environments. Limitations and recommendations for future research are discussed.

## Introduction

*How can we support effective learning?* Answering this question is of utmost practical and scientific interest, as it is key for successful educational and work performance. In times of digital transformation—recently boosted through the challenges occurring from the COVID-19 crisis—effective learning with digital tools and new digital classroom settings gained in importance (Marinoni et al., 2020; Yan et al., 2021). Especially digital videos were recognized by educational institutions to be a promising opportunity to tackle challenges of such settings since they can be provided asynchronously and remotely (Noetel et al., 2021) and have been shown to foster learning (e.g., Tiernan, 2015).

**Fig. 1:**
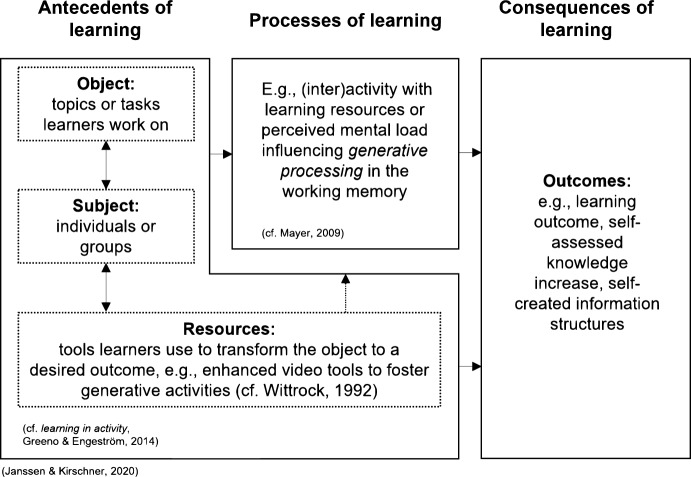
The relationship between antecedents, processes, and consequences of learning, based on situative approaches. *Note*. Explanations to supplement or clarify information in the image. Adapted from “Applying collaborative cognitive load theory to computer-supported collaborative learning: towards a research agenda” by J. Janssen and P. A. Kirschner, 2020, Educational Technology Research and Development, 68(2), p. 790. CC BY 4.0

Nowadays, *enhanced video-based environments* provide *video tools* that allow learners not only to *watch* videos, but to *interact* with the video materials and re-structure them according to their own needs (e.g., Chambel et al., 2006; Zahn et al., 2005). In other words: videos can be used in more flexible and creative ways. For instance, learners can use *annotations* to place their own comments and remarks or self-written summaries into videos, or they can add *hyperlinks* to connect video objects to additional pieces of information thereby creating their own (new) information structures. Moreover, learners can not only work individually, but also collaboratively in such enhanced video-based environments – sharing their annotations and hyperlinks and discuss them with others (peers, colleagues, teachers) or edit the material in learning groups (e.g., Chambel et al., 2006; Goldman, 2007; Sauli et al., 2018; Zahn et al., 2012). In this sense, video tools can be seen as socio-cognitive tools for learning that afford advanced learning activities like “analyze”, “evaluate” or “create” according to Bloom`s taxonomy (Anderson & Krathwohl, 2001; Krathwohl, 2002). However, there is no consensus in the literature on whether and how enhanced tools really support individual and collaborative learning or are rather cognitively overwhelming (Evi-Colombo et al., 2020; Sauli et al., 2018). A *holistic* view on learning might shed light into this question. Situative approaches from cognitive research on learning and computer-supported collaborative learning (CSCL) suggest to study learning by a simultaneous consideration of different aspects that shape learning (Greeno & Engeström, 2014; Janssen & Kirschner, 2020): *antecedents* of learning, such as subjective conditions (individuals or groups), objects (topics and tasks learners work on), and resources (e.g., tools learners use to transform the object to a desired outcome), *processes of learning*, and *consequences of learning*. In Fig. 1, we adapted the model of *the relationship between antecedents, processes, and consequences of collaboration* by Janssen and Kirschner (2020, p.790) and integrated further concepts provided by Greeno and Engeström (2014, *learning in activity*), Wittrock (1992, *generative learning*) and Mayer (2009, *multimedia learning*). These concepts are explained in more detail below.

In the present article, we report on our research systematically investigating in a controlled laboratory experiment how specific resources (video tools) and subjective conditions of learning (individual or collaborative setting) may interact and influence processes and consequences of learning. Precisely, the effects of different enhanced video tools (*Tool-use*: annotations vs. hyperlinks)—alongside with a control group (no *Tool-use*)—and different social learning *Settings* (individual vs. collaborative learning in dyads) on learning processes (i.e., learning activity and cognitive load) and outcomes (i.e., generative learning product and conceptual understanding) were analyzed. In the following sections, we introduce related research on enhanced video-based learning and on individual and collaborative learning. The introduction closes with the aims of the present work.

## Literature review

### From learning with videos to enhanced video tools

Videos have been used to support learning in many domains as they allow to depict objects, situations, and movements (also those that cannot easily be watched with naked eyes) and are able to visualize abstract information processes (Overbaugh, 1995). Even before the COVID-19 pandemic, the use of videos had a long tradition in educational institutions around the globe (for a review see Poquet et al., 2018). However, the rather passive viewing of videos—although it seems very easy (Salomon, 1984)—can result in little engagement, mental effort or reflection of learners, which may impede the construction of their own understanding of a topic (Shin et al., 2018). Thus, the possibility for learners to *interact* with the video material was emphasized to be of crucial importance (Hasler et al., 2007). For example, by using *basic video control tools*, such as play, pause, or rewind, learners are able to actively engage and interact with the material and, thus, to adapt the learning information to their own cognitive needs with positive effects for learning (e.g., Schwan & Riempp, 2004). Or, as another example, *in-video quizzes* afford that learners answer questions that directly appear during video watching (Cattaneo et al., 2018) and, thus, self-assess their own knowledge or discover still existing knowledge gaps (Panadero et al., 2017) with positive effects on learning performance (Haagsman et al., 2020; Rice et al., 2019), engagement (Cummins et al., 2016), and motivation (Leisner et al., 2020).

Today, *enhanced video-based environments* go beyond these types of interaction and instead allow learners to use *enhanced video tools* to actively create their own enriched information structures and thereby generate meaning (for an overview, see Schwartz & Hartman, 2007, for *hypervideo* see Chambel et al., 2006 and Zahn, 2017). According to Bloom’s taxonomy (Krathwohl, 2002), such an active creation of own learning structures is important, since knowledge acquisition is not only a product of “understanding”, but also of "creating" (see also *learning through design approach*: Kafai & Resnick, 1996). This is in line with Wittrock's (1992) model of generative learning and Mayer’s (2009) select-organize-integrate (SOI) model, suggesting that *generative activity* also promotes generative processing in the working memory, i.e., the organizing and integrating of new learning material with prior knowledge (see Fig. 1, cf. Fiorella & Mayer, 2016). More specifically, according to Fiorella and Mayer (2016, p. 717), “generative learning is the process of transforming incoming information (e.g., words and pictures) into usable knowledge (e.g., mental model schemas)” by making either internal or external connections. Learners make internal connections when they make links between different elements of the material to be learned and external connections when they link the material to be learned to existing knowledge.

Examples of supporting students' generative learning include classroom activities such as summarizing and mapping (for an overview, see Fiorella & Mayer, 2016). Summarizing asks students to select relevant new information and put it into their own words, relating this new information to their prior knowledge. Mapping involves linking content from different sources of information and making associations between related concepts. In order to prevent students from being cognitively overwhelmed, it is advisable to provide support tools (Fiorella & Mayer, 2016). Investigating *different* examples of such study activities have been shown to affect learning in *different* ways. For example, Ponce and Mayer, (2014) investigated the effects of note-taking and a graphic organizing tool and found different effects on reading comprehension.

In our research we compare different examples of how specific tools support generative learning activities in video-based learning: functions for *diving on video* that enable users to create own points of view onto a source video and comment on these e.g., for guided noticing (Pea, 2006; Pea et al., 2004) or *annotations*, i.e., self-written notes or *summaries* added to a video (Chiu et al., 2018), and *hyperlinks*, i.e., supplementary material that includes predefined texts or pictures (Meixner, 2017; Zahn, 2017; Zahn et al., 2005), which we understand as a graphical form of mapping.

Enhanced video tools have received a growing interest in education (Noetel et al., 2021) and research reported overall positive effects on learning (for a review, see Evi-Colombo et al., 2020). For example, learners were found to perform better on knowledge tests when they used annotations to learn about first aid (Chiu et al., 2018) or physics (Delen et al., 2014) than when they learned with common video material without enhanced tools. Moreover, rare existing research on hyperlinks (cf. Evi-Colombo et al., 2020) has shown that the creation of own hypervideo structures supports learning of complex history topics (Zahn, 2017; Zahn et al., 2005). Findings from these empirical studies reveal that digital design tasks with video tools were generally effective for learning and applicable to diverse regular in-class, face-to-face and online learning settings (cf. Zahn, 2017). Overall, students’ knowledge and skills significantly increased while significant tool effects were evidenced: An enhanced video tool with a selection function (diving = virtually cutting out video objects from a video sequence, see Pea et al., 2004) proved superior to a control condition (simple video player and text editing tool) concerning design performance, content learning and visual skills acquisition. Qualitative analyses illustrated how students used specific video tool functions for support of guided noticing and joint elaboration of specific content items. However, many studies also reported conflicting results (for an overview, see Sauli et al., 2018). Thomas et al. (2016), for instance, found that learners who used annotations to learn a history topic underperformed learners who learned with common videos. Merkt et al. (2011), who investigated the effects of a common video, an enhanced video with a table of contents, and an illustrated textbook, also found that learning with a common video was more useful to learn history topics than learning with an enhanced video. They argued that performing so-called micro-level activities (resulting from the use of basic video control tools) is unproblematic for learning, while performing macro-level activities (resulting from the use of enhanced tools) caused problems for learners.

From reviewing the empirical results, we assume that general statements about the effectiveness of enhanced tools are still difficult for the following reasons. First, video tools differ in how they support learning, which, in turn, is reflected in learning outcomes. For example, the act of writing promoted by annotation tools stimulates the cognitive processes of reflecting, organizing, and integrating of information (Lawson & Mayer, 2021), which, in turn, supports the understanding and evaluation of individual concepts and the creation of own (new) ideas (Zahn et al., 2012). Hyperlinks, in contrast, support the integration of related pieces of information and their re-structuring, which promotes the understanding of interrelations between concepts (Chambel et al., 2006; Rickley & Kemp, 2020; Stahl et al., 2006; Zahn, 2017). These examples illustrate the importance of considering different impacts on learning processes and outcomes when examining the effects of enhanced tools and their affordances for learning. Second, enhanced tools need to be clearly instructed and included as a *necessary part* of the learning task (Rice et al., 2019; Zahn et al., 2005). So far, however, enhanced tools have often been investigated rather as *optional supporters* for learning. Previous research suggests that learners may not develop appropriate learning strategies to use optional tools for learning (Merkt et al., 2011) and that they may cognitively overwhelm learners rather than support them in effective learning (Krauskopf et al., 2014).

In consequence, direct comparisons of enhanced video tools in systematic research are needed that could help to explain and predict how *different* tools might afford *different* learning activities under concrete learning conditions and in specific task contexts—with important implications for their use in educational practice (see also, Ponce & Mayer, 2014).

Concerning task context, Zahn (2017) developed a framework and new scope of using advanced video collaboration tools together with the paradigm of *digital design for learning.* The author explains the cognitive and collaborative processes involved *in learning through designing one`s own information structures with video tools* based on established cognitive and collaborative problem solving models (e.g., Goel & Pirolli, 1992; Lowry et al., 2004) and constructionism (cf. Kafai & Resnick, 1996).

Concrete learning conditions include both individual and collaborative learning—and video tools have been considered for both ways (e.g., Chambel et al., 2006; Pea, 2006; Zahn, 2017). The focus in these studies has been on *specific functions* that afford either *cognitive activities* in individual learning (e.g., relating concepts by annotations or cognitive maps, Chambel et al., 2006; Rich & Trip, 2011; Stahl et al., 2006) or *socio-cognitive* activities in collaborative knowledge building scenarios (e.g., sharing annotations and comments, jointly developing links, and discussing related contents, Pea et al., 2004; Stahl et al., 2006; Zahn, 2017; Zahn et al., 2010). The effects of such functions on learning outcomes have—to the knowledge of the authors—not been studied intensively empirically and comparatively—recent research has focused more on learning processes, such as cognitive load (Van Sebille et al., 2018), or intentions that determine when learners use such tools (Mirriahi et al., 2021). Here, tool effects were proven (for a summary, see Zahn, 2017) but more research is still needed.

In sum, we conclude that direct comparisons of different *task-relevant* usage of enhanced video tools in systematic research could provide new original results for better insights into the conditions under which they can effectively support learning. This research might also provide new knowledge about possible interactions between video tool functions and learning conditions which would have implications for educational practice. We further consider a simultaneous investigation of the effects of enhanced tools on learning processes *and* learning outcomes, following previous approaches (see Fig. 1, c, f. Greeno & Engeström, 2014; Janssen & Kirschner, 2020) crucial. Accordingly, incorporating investigations of learning processes help to understand (1) how learners use enhanced video tools for learning (i.e., micro- and macro-level learning activities, cf. Merkt et al., 2011) both individually or collaboratively and (2) how these tools influence learners’ cognitive load (Rice et al., 2019; Zahn, 2017). Furthermore, both *generative learning* (e.g., Fiorella & Mayer, 2016; Kafai & Resnick, 1996; Krathwohl, 2002) and *conceptual understanding* should be considered when developing measures to assess learning outcomes to account for different supportive roles of video tools and functions in the light of concrete learning goals (Stahl et al., 2006; Zahn et al., 2012).

### Individual vs. collaborative video-based learning

Although computer-supported collaborative learning (CSCL) has received considerable attention in the last decades—especially in higher education (see e.g., Janssen & Kirschner, 2020; Jeong et al., 2019)—and research on *video-supported collaborative learning* was added to this scientific knowledge (Zahn, 2017; Zahn et al., 2010, 2012) it is still rare (Ramos et al., 2019). Especially, direct comparisons between different social conditions (i.e., individual and collaborative learners) would be important in the research field and for educational practice. Related research on learning with animations, for instance, did such comparisons and found that collaborative learning is superior to individual learning in problem solving (Kirschner et al., 2011; Retnowati et al., 2016), retention, interpretation, explanation (Bol et al., 2012), and transfer tasks (Rebetez et al., 2010). Rebetez et al. (2010) for example, examined the impact of different multimedia instructions that varied regarding their level of interactivity (static vs. animated material) on learning a science topic and found that participants who learned with animations were overall superior in retention tasks, but only collaborative learners benefited from animations in transfer tasks. In a study by Kirschner et al. (2011), the authors examined the impact of different instructional formats (worked examples vs. equivalent problem solving) on individual and collaborative learning outcomes in biology and could show that studying worked examples was better performed by individuals but problem solving tasks were easier for collaborative learners. A more recent study by Liao et al. (2019) further found that collaborative learning combined with instructional videos supports learning achievement and reduces extraneous load in a digital game-based learning context.

In sum, research on animations and game-based learning indicates that collaborative learning fosters learning outcomes for complex tasks with important implications for educational practice. Yet, the possible effects of individual and collaborative learning in enhanced video-based learning still have to be investigated. In the present work, we aim to fill this gap.

### Aim of the present study

Given the high and still growing relevance of videos in educational institutions (Noetel et al., 2021), we aim to tackle the research gaps described above and contribute new original data. In doing so, we follow previous approaches (Greeno & Engeström, 2014; Janssen & Kirschner, 2020) and pursue a holistic approach by systematically and simultaneously examining different antecedents, processes, and consequences of learning in a co-located face-to-face context. According to the literature described above, we assume that different video tools can be used to foster different generative activities, which, in turn, should support learning and conceptual understanding in different ways in individual vs. collaborative learning situations and with possible interaction effects. We use two representative video tool functions (annotation and hyperlinks) that are common representatives of enhanced video-based learning environments in education (Cattaneo et al., 2018; Chambel et al., 2006; Rich & Trip, 2011; Stahl et al., 2006; Zahn, 2017; Zahn et al., 2005, 2010) and have been discussed with respect to their possible functions for learning before while the learning activities they *could* possibly afford were specified in detail (Chambel et al., 2006; Zahn, 2017; Zahn et al., 2010). We connect up to this research by investigating such *possible* effects.

For the purpose of the present study, we pursue the following research question: How does learning in different *Tool-use* (annotations vs. hyperlinks vs. control) and different *Setting* conditions (individual vs. collaborative learning in dyads) impact learning processes (H1), outcomes (H2), and their relations (H3)? The following hypotheses are derived from this: first, regarding the effects on learning processes, we expect (H1a) differences in *Tool*-*use* and (H1b) *Setting*. Second, regarding *learning outcomes*, we expect (H2a) an overall knowledge gain after learning over all conditions, (H2b) differences in *Tool-use*, and (H2c) a superiority of collaborative over individual learning (i.e., *Setting*). Third, we exploratory investigate (H3) if learning processes can function as possible mediators between the independent variables *Tool-use* and *Setting* and the dependent variables of learning outcome.

## Methods

### Participants and study design

**Fig. 2:**
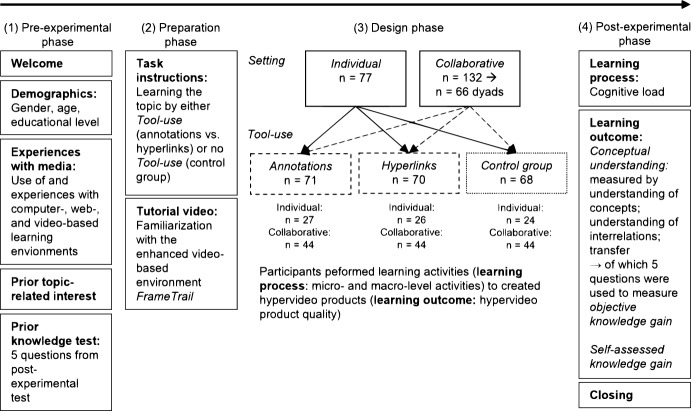
Study design and procedure

Overall, 209 participants were part of this experiment (74.6% female, *M* = 24.30, *SD* = 6.7) which took place in a controlled laboratory setting at a Swiss University. Figure 2 includes detailed information on the study design and sample size. The ethical standards were met as is confirmed by the ethical review board of our institution. Each participant received either course credits or 20.- Swiss francs for participation. Participants were randomly assigned to the experimental conditions of a 3 × 2 study plan. The first factor (*Tool-use*) determined whether participants were allowed to use enhanced tools and/or which tools they were allowed to use to perform the task: *annotations* of self-written summaries, *hyperlinks*, or no option to use enhanced tools (no *Tool-use* = control group). The second factor concerned the social learning *Setting*: participants learned either alone (individual condition) or collaboratively in dyads (collaborative condition). We studied dyads rather than larger groups for four reasons: first, as participants were learning together in front of a desktop computer with one screen and one interaction device, we intended to ensure the best possible collaborative learning situation in terms of accessibility for interaction and visibility. This situation is close to natural settings often used in real educational school-based or higher education. Second, as we focused on comparing individual and collaborative learning, and as research on the effect of group size on learning outcomes is inconsistent here (Caulfield & Caroline, 2006; Kim et al., 2020), we started with dyads for reasons of feasibility in the present project. Knowing that collaborative processes in dyads vs. small groups of three or four are not always the same, we still expect our results to be extremely relevant for some settings and more importantly that they will provide a foundation for future investigation. Third, because of the nature of our task, which aims at learning about a natural science topic (cell biology) and not at learning to discuss conflicting or multiple perspectives (e.g., in politics) we do not assume that the results would have been largely different with larger groups. Last, studying dyads also facilitates methodological evaluation (e.g., in relation to video analysis and comparisons with previous related studies that were also conducted with dyads, see Zahn, 2017).

### Procedure

The experimental procedure lasted approximately one and a half hours and followed four phases (see Fig. 2). In the pre-experimental phase, participants were welcomed and asked to complete questions about their demographics (gender, age, educational level), their prior experience with digital media for learning, prior self-assessed (what persons think they know) and objective knowledge (what persons actually know), and prior topic-related interest. Next, in the preparation phase, participants were instructed to their task to learn about *synaptic plasticity* for a post-experimental questionnaire either by using enhanced tools (*Tool-use*: annotations vs. hyperlinks) or without the possibility to use enhanced tools (control group) and were asked to familiarize themselves with the environment by a tutorial video. In the design phase, participants were then asked to accomplish the task either individually or in dyads working on a shared desktop computer (= *Setting*). Participants in the *Tool-use* conditions were additionally asked to use enhanced tools to create a high-quality hypervideo product that should also help other students to learn in the future. Participants in the collaborative settings were instructed to discuss the material carefully with their learning partner to learn the material and to fulfill their task (e.g., formulate summaries and insert them to appropriate video sequences). Participants in the control group were asked to watch the video considerately. The design phase took on average 35.04 min (*SD* = 16.8). Participants could invest as much time as they needed. This should ensure that they were able to fully understand the content, to compensate for effects of cognitive load, and to complete the given task. In the post-experimental phase participants were finally asked to complete the post-questionnaires before they were thanked and released.

### Materials

**Fig. 3:**
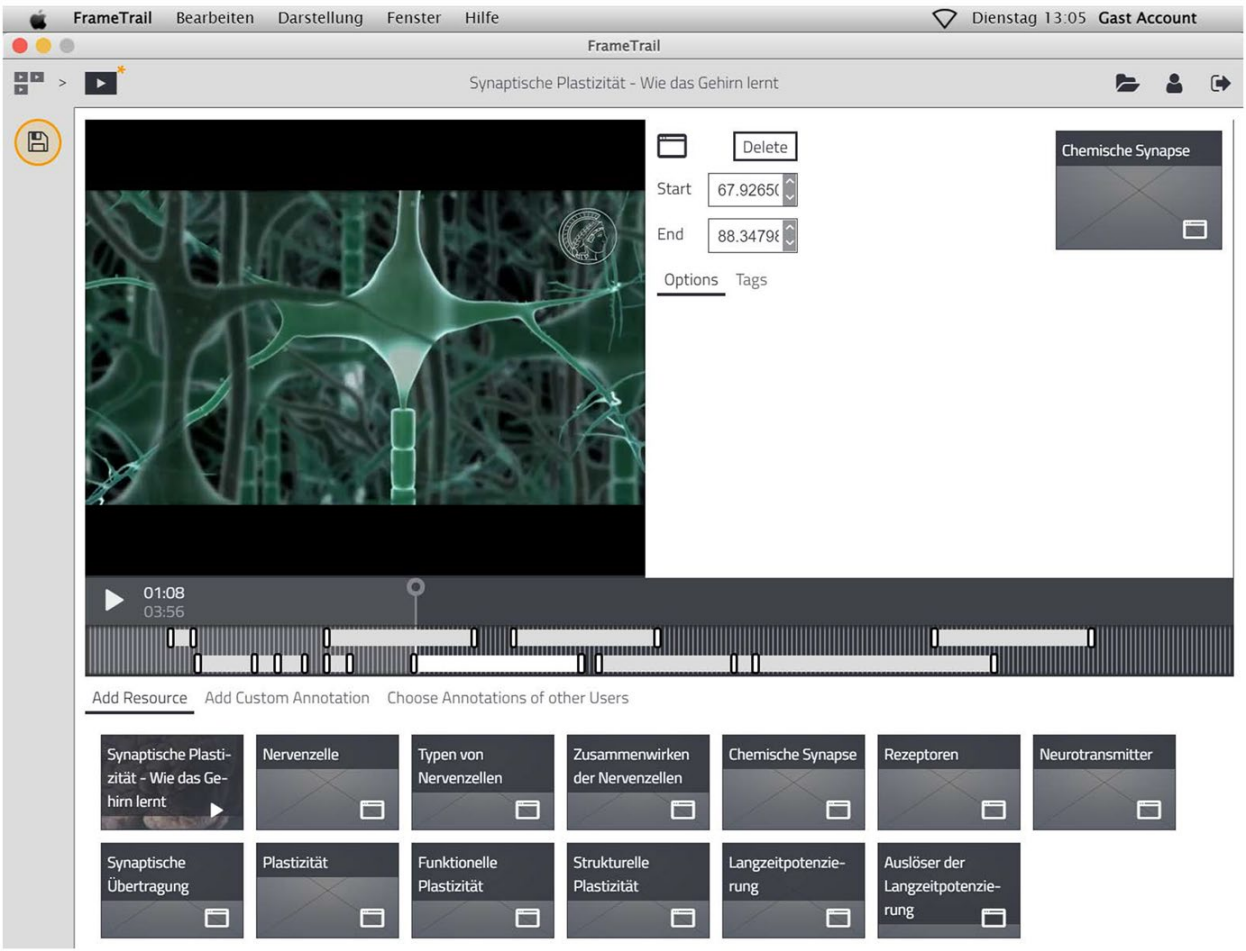
Illustration of the enhanced video-based environment *FrameTrail*

The exemplary video used as learning material for the experiment addressed the neuroscience topic *synaptic plasticity of the human brain*. It was originally produced as high-quality instructional learning material by the Max Planck Society and lasts 3:56 min. The video was embedded in the enhanced video-based environment *FrameTrail* (see Fig. 3). This environment additionally included topic-related in-depth information in form of prepared text snippets directly available below the video*.* These texts were developed together with an expert in the field of neurobiology. While participants in the annotation condition were asked to read these texts and then write own summaries (i.e., annotations) to add them into the video, participants in the hyperlinks condition could grab these text snippets and add them directly into the video at appropriate places (per drag and drop). In both conditions, the display time of the hyperlinks or annotations could be manually changed to adapt the additional learning material to the relevant part of the video. Participants in the control group were asked to simply watch the video and read the text snippets (i.e., common video learning).

### Measures

Measures and scores of the dependent variables on learning processes and outcomes are summarized in Table 1.

**Table 1:** Measures and scores of dependent variables on learning processes and outcomes

Dependent variables	Measures/items	Scores/scales
Learning processes		
*Learning activity*		
Micro-actions (basic video control tools)	Logs of play, pause, skipping forward, skipping backward	Relative values of actions per min
Macro-actions (enhanced video tools)	Logs of adding, deleting, or changing display time of annotation/hyperlink and changes of annotation text	
*Cognitive load*	One-item scale: “Please estimate how easy or how difficult you found the learning material”	1 (very easy)–7 (very difficult)
Learning outcomes		
*Hypervideo product quality (HPQ)*	Knowledge transforming	*Grading system:* 1 (lowest grade)–6 (top grade) *Information structuring*: max. 300 points
	Information structuring	
	Formal criteria	
	Knowledge development	
	Overall impression	
*Conceptual understanding*	Understanding of concepts, 8 questions (2 included in prior knowledge test)	8 points
	Understanding of interrelations, 8 question (2 included in prior knowledge test)	8 points
	Transfer knowledge, 4 questions (1 included in prior knowledge test)	4 points
*Self-assessed knowledge gain*	One-item scale: “How much do you think your knowledge of synaptic plasticity has improved?”	1 (not at all)–5 (very much)

To measure *learning processes*, we, on the one hand, collected participants’ *learning activities* by using log-file protocols provided by *FrameTrail*. These files included information about video interaction for each individual and dyad (i.e., actions such as clicking on the “play” button). According to previous research (Delen et al., 2014; Merkt et al., 2011), learning activity was measured by distinguishing between (1) micro- and (2) macro-actions. While micro-actions included logs of interactions with basic video control tools, i.e., play, pause, skipping forward and backward, macro-actions covered interactions with enhanced tools, i.e., adding or deleting hyperlinks or annotations, changes of the display time on the video timeline, and changes of self-written annotation texts. The fact that participants learned at their own pace was reflected in a spread of variance for both the absolute learning time (annotation: *M* = 47.46 min, *SD* = 16.2; hyperlinks:* M* = 35.51 min, *SD* = 11.6; control group: *M* = 21.85 min, *SD* = 11.2) and the absolute frequencies of performed micro- (annotations: *M* = 65.15, *SD* = 35.1; hyperlinks: *M* = 110.13, *SD* = 47.3; control group: *M* = 3.71, *SD* = 7.2) and macro-actions over all participants (annotations: *M* = 86.04, *SD* = 48.6; hyperlinks: *M* = 48.81, *SD* = 22.2; control group: no macro-actions according to the learning task). We thus considered relative values of these actions (absolute performed actions divided by total learning time in minutes) to take account of varying total learning times. Although analyses for both absolute and relative values were conducted, we focus on relative values hereafter. On the other hand, we measured *cognitive load*, according to previous definitions of the concept (De Jong, 2010; Paas, 1992), with the one-item scale “Please estimate how easy or how difficult you found the learning material” from 1 (very easy) to 7 (very difficult) according to Kalyuga et al. (1999). Note, we used this one-item scale as it does not only extensively measure subjective perceived task difficulty but is also widely and successfully used in research on cognitive load (Brünken et al., 2010; De Jong, 2010). We, moreover, refer to previous research on the effectiveness of one-item scales (Gogol et al., 2014).

To collect *learning outcomes*, we were guided by the above-mentioned literature (Fiorella & Mayer, 2016; Krathwohl, 2002; Wittrock, 1992) and investigated both (1) *generative learning product*, by measuring the quality of self-created hypervideo products, and (2) *conceptual understanding* of the topic. First, *hypervideo product quality* (HPQ) was measured by the ratings of two experts with 10% of the products rated by both experts (Cronbach’s alpha: 0.96). The experts evaluated several quality indicators based on a grading system developed for this study: (1) knowledge transformation (correctness of content and use of own words instead of “copy/paste”), (2) information structuring (correct placement the enhanced tools, completeness of content, meaningful change of display time), (3) formal criteria (text length, correct grammar, use of titles), and (4) knowledge development (additional information provided by the learners based on their prior knowledge). Additionally, experts rated (5) an overall impression of the products. To compare HPQ of the annotation and hyperlink condition, we developed a weighted grading system (total points achieved * 5 divided by max. score) from 1 (lowest grade) to 6 (top grade). Note, while all categories could be addressed in the annotation condition, the hyperlink condition could only be evaluated via (2) information structuring and (5) overall impression. Thus, we conducted separate analyses with “information structuring”, which was equally measured in both conditions. A maximum of 300 points could be scored (see Table 1).

Second, *conceptual understanding* was measured using a post-experimental questionnaire including eight questions that addressed ‘understanding of concepts’ (6 MC- and 2 short answer questions, e.g., “what are vesicles?” referring to understanding the concept of vesicles), eight questions that addressed ‘understanding of interrelations’ between concepts (6 MC- and 2 short answer questions, e.g., “what role do calcium ions play in synaptic transmission?” referring to an understanding of the interrelation of calcium ions and synaptic transmission), and four questions that addressed ‘transfer knowledge’ (3 MC- and 1 short answer questions intended to measure the ability to transfer learned information to other situations or circumstances, see Rebetez et al., 2010). Learners received one point for a correct and zero points for an incorrect answer. The Cronbach’s alpha of the full test was 0.76. Moreover, we randomly selected two questions each of ‘understanding of concepts’ and ‘understanding of interrelations’ and one ‘transfer knowledge’ question that we integrated in the pre-experimental questionnaire. These five questions were used to check for between-group comparisons and to measure *objective knowledge gain*.

Third, we collected *self-assessed knowledge gain* using a post-experimental one-item scale. Participants were asked to specify how much they think their knowledge about synaptic plasticity has improved after learning from 1 (not at all) to 5 (very much).

### Data analysis

Regarding the data analysis of the present study, the following should be noted: the first focus of the present study relied on the impact of different enhanced tools on learning processes and outcomes. For this reason, we included measures that could not be collected for the control group (i.e., macro-actions and HPQ). Consequently, the control group was not considered in all analyses. Analyses that could be conducted with the control group were performed both with and without the control group. The second focus of this study relied on the impact of different social settings on learning processes and outcomes. Hence, 132 participants learned and performed the task in dyads on a shared desktop computer (= 66 dyadic settings). As a result, measures on learning activity and HPQ were only available at the group level while other measures were available at the individual level. Hence, to compare the different settings with analyses of variance, data from learners in groups were aggregated (by averaging group means). With this, we refer to previous research, suggesting interdependence of students working in one team (Kenny et al., 2006). Still, we additionally performed multilevel analyses by using HLM (Raudenbush & Bryk, 2002) and found similar effects. Moreover, standard deviations between individual and collaborative learners were found to be relatively similar (see Tables 2, 3, 4, 5). Therefore, we considered our measures to be independent.

To test our hypotheses, we used the following analyses. First, regarding H1 (learning processes), we conducted a two-way multivariate analysis of variance (MANOVA) with micro- and macro-actions (i.e., learning activity) as dependent variables. The control group was not considered. Moreover, a two-way analysis of variance (ANOVA) was conducted with cognitive load as dependent variable.

Second, regarding H2 (learning outcomes), we conducted a two-way repeated-measures ANOVA for objective knowledge gain (H2a). Furthermore, two similar two-way ANOVAs with either HPQ or information structuring as dependent variables were conducted (H2b, H2c). The control group was not considered. Also, a two-way MANOVA was performed with the variables ‘understanding of concepts’, ‘understanding of interrelations’, and ‘transfer knowledge’ (i.e., conceptual understanding) as dependent variables. Last, we conducted a two-way ANOVA with self-assessed knowledge gain as dependent variable.

Third, we intended to exploratory investigate effects of learning processes as pre-supposed mediators between the independent variables *Tool-use* and *Setting* and the dependent variables on learning outcomes in H3. Therefore, we checked for assumptions to conduct mediation analyses, on one hand, provided by the findings demonstrated in our analyses on learning processes (H1) and outcomes (H2), and, on the other hand, by running a series of bivariate correlations (Pearson) between the mediators and the dependent variables (Baron & Kenny, 1986). The assumption checks are discussed separately in the results section below. The mediation analyses were then conducted using the regression-based approach for conditional process modeling using the SPSS-macro PROCESS (Hayes et al., 2017). The control group was not considered in these analyses because no data on macro-actions and HPQ were available. For all analyses, requirements were checked in advance referring to relevant literature (e.g., Blanca et al., 2017; Finch, 2005; Kenny et al., 2006).

## Results

### Between-group comparisons and manipulation check

The analyses for gender (chi-square test), age, educational level, prior experience with digital media for learning, prior self-assessed knowledge, and prior interest in the learning topic (ANOVAs) yielded no significant differences in *Tool-use* (annotations vs. hyperlinks vs. control, *p* > 0.10) nor *Setting* (individual vs. collaborative, *p* > 0.10). Hence, groups were comparable on these variables. For manipulation check, the hypervideo products of the annotation and hyperlink condition were compared by an expert using log data of learners’ interaction to confirm that participants in the *Tool-use* conditions only performed the task they were assigned to. Results revealed that participants in the annotation condition only added annotations, participants in the hyperlink condition only hyperlinks, and that no enhanced tools were used in the control group.

### Impact of *Tool-use* and *Setting* on learning processes (H1)

Learning activity (see Table 2): MANOVA revealed a statistically significant effect for *Tool-use* (as expected in H1a), *F*(2,88) = 41.709, *p* < 0.01, partial η^2^ = 0.487, Wilk’s Λ = 0.513. Additionally conducted post-hoc ANOVAs revealed that participants that learned with annotations performed significantly *more* macro-actions (annotation: *M* = 1.75, *SD* = 0.6; hyperlink:* M* = 1.45, *SD* = 0.7, *F*(1,89) = 1.978, *p* = 0.033, partial η^2^ = 0.050), and significantly *fewer* micro-actions than participants that learned with hyperlinks (annotation: *M* = 1.41, *SD* = 0.7; hyperlink: *M* = 3.22, *SD* = 1.3, *F*(1,89) = 74.116, *p* < 0.01, partial η^2^ = 0.440). No significant effect was found for *Setting* (H1b, *p* > 0.10).

Cognitive load (see Table 3): results yielded significance for *Tool-use, F*(1,92) = 9.000, *p* = 0.003, partial η^2^ = 0.089 (as expected in H1a), indicating that learners in the annotation condition perceived a significant *lower* cognitive load (*M* = 3.75, *SD* = 1.2) than learners in the hyperlink condition (*M* = 4.49, *SD* = 1.2). No effects were found for *Setting* (H1b, *p* > 0.10). A similar analysis that considered the control group revealed no additional significant results (*p* > 0.10).

**Table 2:** Descriptive data (Mean, SD) of learning activity

*Tool-use*	Annotations		Hyperlinks	
*Setting*	Individual	Dyad	Individual	Dyad
Micro-actions	1.41 (.7)	1.42 (.7)	3.38 (1.5)	3.04 (.9)
Macro-actions	1.83 (.7)	1.66 (.6)	1.50 (.7)	1.40 (.7)

**Table 3:** Descriptive data (Mean, SD) on cognitive load

*Tool-use*	Annotations		Hyperlinks		Control group	
*Setting*	Individual	Dyad	Individual	Dyad	Individual	Dyad
(Scale from 1 to 7)	3.73 (1.3)	3.77 (1.2)	4.58 (1.3)	4.39 (1.0)	3.83 (1.2)	4.02 (1.0)

### Impact of *Tool-use* and *Setting* on learning outcome (H2)

Hypervideo product quality (see Table 4): results revealed a significant main effect for *Tool-use* (as expected in H2b), *F*(1,88) = 24.414, *p* < 0.01, partial η^2^ = 0.216, *d* = 1.01, indicating that learners in the hyperlink condition produced hypervideo products of higher quality (*M* = 4.96, *SD* = 0.4) than learners in the annotation condition (*M* = 4.47, *SD* = 0.5). Moreover, a marginal significant effect for *Setting* was found, *F*(1,88) = 3.605, *p* = 0.061, partial η^2^ = 0.039, *d* = 0.36, indicating that dyads (*M* = 4.82, *SD* = 0.6) slightly outperformed individuals (*M* = 4.63, *SD* = 0.5), as expected in H2c. An analysis with information structuring revealed similar results: a significant effect for *Tool-use*, *F*(1,88) = 52.36, *p* < 0.01, partial η^2^ = 0.373, *d* = 1.47, with a superiority of the hyperlink condition (hyperlink: *M* = 243.16, *SD* = 25.0; annotation: *M* = 199.99, *SD* = 33.3), and a marginally significant effect for *Setting, F*(1,88) = 3.485, *p* = 0.065, partial η^2^ = 0.038, *d* = 0.32, with higher scores for dyads (dyads: *M* = 228.16, *SD* = 38.5; individuals: *M* = 216.68, *SD* = 33.9). These results, too, confirm H2b and c.

Conceptual understanding (see Table 5): as expected in H2a, results yielded significance,* F*(1,93) = 119.47, *p* < 0.001, Wilks’ Lambda = 0.438 (*M*_prior_ = 2.44, *SD*_prior_ = 1.3; *M*_post_ = 3.91, *SD*_post_ = 1.0), indicating an objective knowledge gain after learning over all conditions. No significant differences were found between the conditions (all *p* > 0.10). An equal analysis considering the control group revealed similar results (knowledge gain: *F*(1,137) = 210.57, *p* < 0.001, Wilks’ Λ = 0.394; *M*_prior_ = 2.47, *SD*_prior_ = 1.3; *M*_post_ = 3.99, *SD*_post_ = 1.0; no differences between the conditions, all *p* > 0.10). Moreover, in contrast to our assumptions in H2b and H2c, no significance differences were found for *Tool-use* or *Setting* on the combined dependent variables ‘understanding of concepts’, ‘understanding of interrelations’, and ‘transfer knowledge’ (all *p* > 0.10). A similar analysis that considered the control group, however, yielded significance for *Tool-use*, *F*(6,270) = 2.87, *p* = 0.010, partial η^2^ = 0.060, Wilk’s Λ = 0.884. Post-hoc univariate ANOVAs revealed significance for the variables ‘understanding of interrelations’, *F*(2,137) = 6.530, *p* = 0.002, partial η^2^ = 0.087, and ‘transfer knowledge’, *F*(2,137) = 4.294, *p* = 0.016, partial η^2^ = 0.059, both indicating that the control group outperformed the two other conditions (Tukey post-hoc analyses: ‘understanding of interrelations’: control > annotation, *p* = 0.022 (Mdiff = 0.91, 95%-CI[0.11,1.71]); control > hyperlinks, *p* = 0.003 (Mdiff = 1.15, 95% CI[0.35,1.96]; *‘*transfer knowledge’: control > annotation, *p* = 0.010 (Mdiff = 0.50, 95% CI[0.10,0.90])). Results on *Setting* were not significant (H2c, *p* > 0.10).

Self-assessed knowledge gain (see Table 5): a significant effect for *Tool-use* was found, *F*(1,93) = 5.907, *p* = 0.017, partial η^2^ = 0.60, indicating (as expected in H2b) that learners using annotations had experienced a higher knowledge gain (*M* = 3.83, *SD* = 0.8) than learners using hyperlinks (*M* = 3.50, *SD* = 0.6). No effects were found for *Setting* (H2c, *p* > 0.10). A similar analysis additionally considering the control group did not reveal further significant effects (*p* > 0.10).

**Table 4:** Descriptive data (Mean, SD) of HPQ and information structuring

*Tool-use*	Annotations		Hyperlinks	
*Setting*	Individual	Dyad	Individual	Dyad
HPQ (grades from 1 to 6)	4.41 (.5)	4.53 (.5)	4.84 (.4)	5.09 (.4)
Information structuring (max. 300 points)	198.15 (33.0)	202.09 (34.4)	234.47 (24.2)	253.04 (22.4)

**Table 5:** Descriptive data (Mean, SD) of conceptual understanding

*Tool-use*	Annotations		Hyperlinks		Control group	
*Setting*	Individual	Dyad	Individual	Dyad	Individual	Dyad
Understanding of concepts (max. 8 points)	5.67 (1.6)	5.71 (1.3)	5.96 (1.3)	5.23 (1.4)	6.38 (1.4)	5.91 (1.1)
Understanding of interrelations (max. 8 pints)	5.07 (1.8)	4.46 (1.4)	4.62 (1.9)	4.48 (1.4)	5.71 (1.7)	5.71 (1.6)
Transfer knowledge (max. 4 points)	2.78 (.9)	2.82 (.8)	3.04 (.9)	2.96 (.8)	3.33 (.9)	3.25 (.6)
Self-assessed knowledge gain (scale from 1 to 5)	3.81 (.8)	3.84 (.6)	3.58 (.7)	3.41 (.4)	3.67 (.8)	3.70 (.7)

### Assumption checks for mediation analyses

Before conducting the mediation analyses, we, first, took a closer look at the results found for H1 and H2. The results showed that no or only marginal effects were found for the independent variable *Setting*. Thus, we subsequently focused on *Tool-use* as independent variable for the following mediation analyses. Second, Pearson correlations were performed with learning process variables (pre-supposed mediators) and variables relating to learning outcome (see Table 6). Significant correlations were found between learning activity and HPQ, information structuring, and self-assessed knowledge gain. Moreover, results revealed that cognitive load positively correlated with ‘understanding of concepts’, ‘understanding of interrelations’, and ‘transfer knowledge’. The analyses further revealed (1) that micro- and macro-actions did not correlate, which is why they were included as separate mediators in the following analyses, (2) that the three dependent variables on conceptual understanding correlated, whereas we combined these variables into the single variable “conceptual understanding” (by sum) for further analyses, and (3) significant correlation between macro-actions and cognitive load, indicating that the less macro-actions were performed by learners, the lower was perceived cognitive load.

**Table 6:** Pearson correlations among mediators and dependent variables (note: aggregated means were used for dyads)

Variables		*M*	*SD*	N	1	2	3	4	5	6	7	8	9
1	Micro-actions	2.33	1.4	93	–								
2	Macro-actions	1.60	0.7	93	− 0.01	–							
3	Cognitive load	4.12	1.2	96	0.16	− .253*	–						
4	Understanding of concepts	5.65	1.4	97	0.06	0.20	− .467**	–					
5	Understanding of interrelations	4.68	1.6	97	− 0.06	− 0.06	− .406**	.454**	–				
6	Transfer knowledge	2.90	0.8	97	0.04	0.03	− .271**	.436**	.366**	–			
7	“Conceptual understanding”	13.23	3.1	97	0.01	0.07	− .502**	.818**	.837**	.666**	–		
8	Self-assessed knowledge gain	3.66	0.7	97	− .280**	.218*	− 0.02	0.03	0.03	0.00	0.03	–	
9	Hypervideo product quality (HPQ)	4.72	0.5	92	.288**	0.08	0.01	0.02	0.13	0.04	0.09	− 0.07	–
10	Information structuring	222.04	36.4	92	.409**	0.05	0.07	− 0.02	0.03	0.05	0.02	− 0.13	.918**

*p < .05

**p < .01

In sum, based on the results found regarding H1 and H2 and the Pearson correlations, the following mediation analyses were conducted with *Tool-use* as independent variable: (1) learning activity (micro-, and macro-actions) as mediator for HPQ and information structuring, (2) learning activity as mediator for self-assessed knowledge gain, and (3) cognitive load as mediator for “conceptual understanding”.

### Mediating effects of learning processes on learning outcomes (H3)

**Fig. 4:**
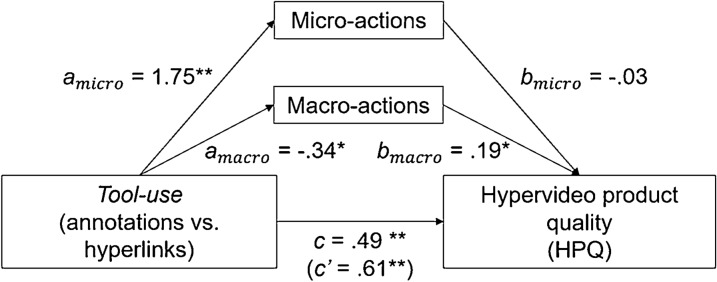
Mediation analysis *Tool-use*, learning activities, and HPQ

**Fig. 5:**
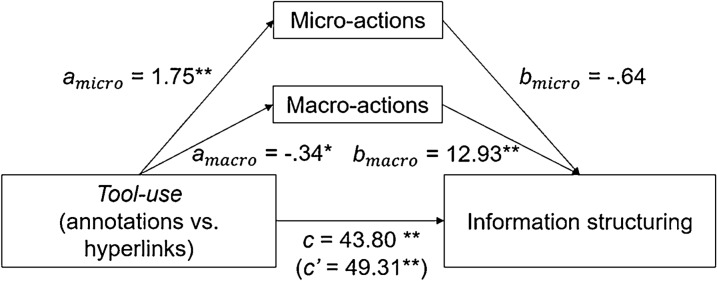
Mediation analysis *Tool-use*, learning activities, and information structuring

Learning activity, HPQ, and information structuring (see Figs. 4 and 5): the mediation analysis with HPQ as dependent variable revealed a significant *c* path, indicating a total effect of *Tool-use* on HPQ (β = 0.49, t = 4.78, *p* < 0.001). Moreover, both *a* paths of learning activity were significant, indicating an effect of *Tool-use* on the performance of micro- (β = 1.75, t = 8.25, *p* > 0.001) and macro-actions (β = − 0.34, t = − 2.56, *p* = 0.012). Moreover, while the *b* path from macro-actions to HPQ was significant (β = 0.19, t = 2.54, p = 0.013) the *b* path for micro-actions was not (*p* > 0.10). Last, the direct effect of *Tool-use* on HPQ controlling for learning activity was still significant (path *c’*; β = 0.61, t = 4.07, *p* < 0.001), suggesting a partial mediation. We found that the relationship between *Tool-use* and HPQ is mediated by the amount of less performed macro-actions, *ab* = − 0.119, 95% CI[− 0.294, − 0.011], but not by the amount of performed micro-actions, *ab* = − 0.091, 95% CI[− 0.389, 0.227]. A mediation analysis with information structuring as dependent variable revealed similar results (macro-actions: *ab* = − 4.381, 95% CI[− 10.115, -0.577]; micro-actions: *ab* = − 1.124, 95% CI[− 10.43, 8.429]).

**Fig. 6:**
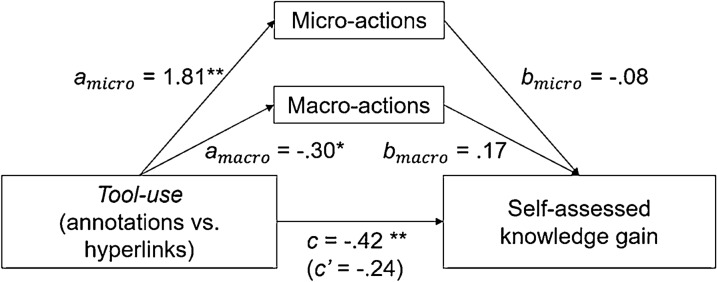
Mediation analysis *Tool-use*, learning activities, and self-assessed knowledge gain

Learning activity and self-assessed knowledge gain (see Fig. 6): the mediation analysis revealed a significant *c* path, indicating a total effect of *Tool-use* on self-assessed knowledge gain (β = − 0.42, t = − 3.24, *p* = 0.002). Furthermore, both *a* paths for learning activity were significant, indicating an effect of *Tool-use* on micro- (β = 1.81, t = 8.45, *p* > 0.001) and macro-actions (β = − 0.30, t = − 2.22, *p* = 0.03). However, the *b* paths and the *c’* path were not significant (*p* > 0.10). This suggests that the relationship between *Tool-use* and self-assessed knowledge gain is not mediated by learning activity.

**Fig. 7:**
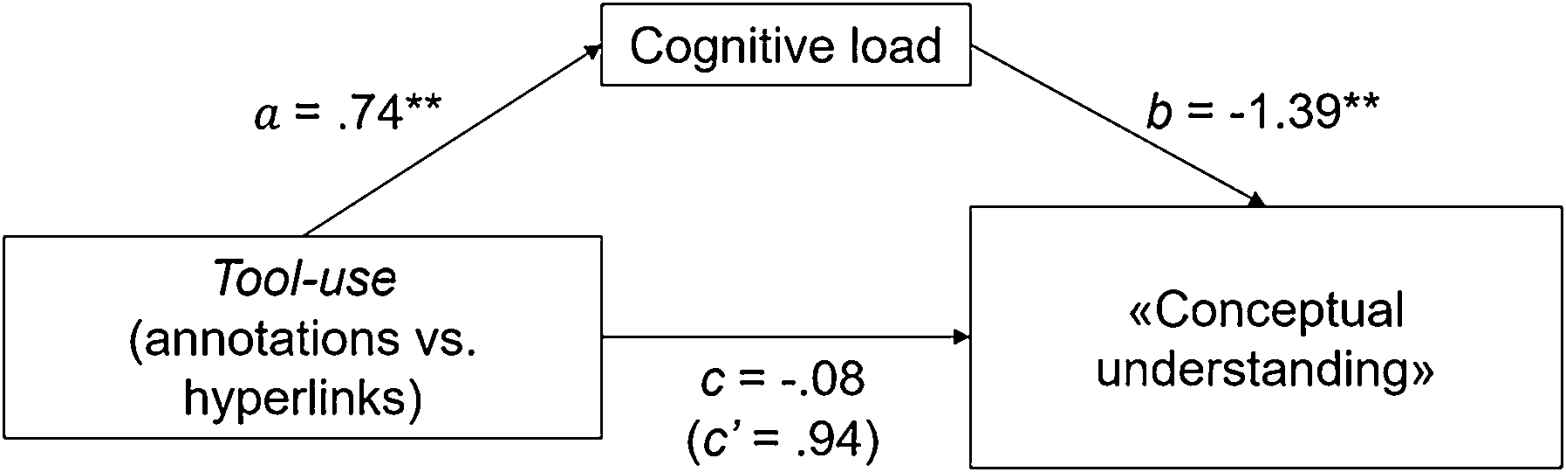
Mediation analysis *Tool-use*, learning activities, and “conceptual understanding”

Cognitive load and “conceptual understanding” (see Fig. 7): the mediation analysis revealed no significant *c* path, indicating that there was no total effect of *Tool-use* on “conceptual understanding” (β = − 0.083, t = − 0.13, *p* > 0.10). However, following previous approaches (Rucker et al., 2011; Zhao et al., 2010), we continued with the analysis. A significant *a* path was found, indicating an effect of *Tool-use* on cognitive load (β = 0.740, t = 3.05, *p* = 0.003). Moreover, the *b* path was found to be significant, indicating an effect of cognitive load on “conceptual understanding” (β = − 1.388, t = − 5.05, *p* < 0.001). Last, the relationship between *Tool-use* and “conceptual understanding” was found to be mediated by cognitive load, *ab* = − 1.027, 95% CI[-1.738, -0.391].

## Discussion

The present work aimed to close current research gaps to the question *how different enhanced video-tools can support generative learning and conceptual understanding in individual and groups?* We intended to provide new original findings on how learning with an interactive video in different *Tool-use* conditions (annotations vs. hyperlinks vs. no *Tool-use*: control group) and in different social *Settings* (individual vs. collaborative learning) impact learning processes (H1) and learning outcomes (H2) in a co-located face-to face-context. Since our results indicate a close association between learning processes and outcomes (H3), we discuss them, first, in terms of *generative learning*, followed by *conceptual understanding*. The discussion closes with limitations and recommendations for future work.

### The impact of *Tool-use *and *Setting* on generative learning (HPQ)

Our results indicate (H2b) that the hypervideo products of learners who were asked to use hyperlinks were of better quality according to experts than of learners who were asked to use self-written annotations. To explain this result, we need to take a closer look at our instrument for measuring hypervideo product quality (HPQ). The instrument considered the different foci of the two *Tool-use* conditions: since learners of the hyperlink condition were asked to learn by expanding the video material with predefined text snippets, their focus was primarily on a meaningful structuring of the material. Therefore, they were primarily evaluated according to the quality of their information structuring. In contrast, learners of the annotation condition were additionally asked to expand the video material with *self-written content*. These learners were, thus, also evaluated according to the quality of their writing activities. The different focus between the conditions was also reflected in different learning activities: more micro-actions but less macro-actions were performed in the hyperlink compared to the annotation condition (H1a). From this we conclude the following: a high quality of information structuring seems to depend on a sparse but target-oriented use of enhanced tools that requires multiple interactions with basic control tools in advance. In other words: learners use basic video control tools (i.e., skipping forward and backward, pressing play and pause) to correctly place and adapt hyperlinks to relevant parts of the video. This is also reflected in our results, (1) indicating that the effect of *Tool-use* on HPQ was partially mediated by the performance of *less* macro-actions (H3) and (2) by a positive correlation of micro-actions with HPQ (see Table 6). Hence, it appears that learners in the hyperlink condition, who were able to focus more on information structuring, were able to create hypervideo products of higher quality, which is in line with previous research (Schwartz & Hartman, 2007; Zahn et al., 2005). In contrast, learners in the annotation condition did not seem to distribute their focus evenly between the two tasks information structuring and writing but focused mainly on the latter. This was reflected in more performed macro-actions and resulted in poorer hypervideo products. Our results further revealed that dyads (marginally) designed hypervideo products of higher quality than individuals (H2c). In line with previous research, we, thus, argue that enhanced environments support socio-cognitive processes (Schwartz & Hartman, 2007) and enable collaborative learners to jointly engage with the material (Sinha et al., 2015).

As stated by earlier approaches on learning though design (cf. Kafai & Resnick, 1996; Krathwohl, 2002) a high quality of own constructed learning material should also foster conceptual understanding, deep processing, and re-organizing of concepts (see also Rickley & Kemp, 2020; Wittrock, 1992). Hence, according to our results, it could be assumed that learners in the hyperlink condition and in collaborative settings should also have higher outcomes in conceptual understanding. However, our results suggest that this is not necessarily the case. This is discussed in the next section.

### The impact of *Tool-use* and *Setting* on conceptual understanding

Our results indicate a general knowledge increase after learning with the enhanced video-based environment over all conditions (H2a). This is in line with previous research on video learning (Evi-Colombo et al., 2020; Poquet et al., 2018) and with research on collaborative learning (Janssen & Kirschner, 2020; Liao et al., 2019). However, we could not find differences in conceptual understanding for *Tool-use* (H2b) or *Setting* (H2c). Our results even demonstrate a superiority of the control group. This result could be explained by the fact that participants in the control group were able to focus exclusively on learning the topic. Thus, the capacity of their working memory may have been higher, leading to better results in conceptual understanding (cf. Baddeley, 2010; Maj, 2020). Moreover, in contrast to previous assumptions (e.g., Krathwohl, 2002; Rickley & Kemp, 2020), we could not show that successful information structuring (high-quality hypervideo products) led to deeper conceptual understanding. More precisely, we could not find a significant relationship between high-quality hypervideo products and conceptual understanding, and we found that learners in the hyperlink condition, who created hypervideo products of higher quality compared to learners in the annotation condition, did not achieve higher results in conceptual understanding. A possible explanation is that the present study focused on short-term memory effects. According to Kassymova et al. (2020), the usage of ‘e-tasks’ support the proper function of the brain’s limbic system that increase neuroplasticity of these parts in the brain that are responsible for long term memory (see also Zull, 2004). Therefore, the effect of constructing own information structures by using enhanced tools on knowledge acquisition could develop over time.

However, when additionally considering learning processes the following picture appears: our results indicate that not learners in the control group, but learners in the annotation condition perceived the lowest cognitive load (H1a). Lower cognitive load was further found to be related with higher outcomes in conceptual understanding and to mediate the effect of *Tool-use* on conceptual understanding (H3). Moreover, our results suggest that lower cognitive load is related to frequently performed macro-actions. Hence, we conclude that an active and frequent use of enhanced tools, which occurs more often in the annotation condition (H1a), can support conceptual understanding, but only when low cognitive load is perceived. In other words: if learners using annotations did not perceive the learning material too complex, annotations could help them build conceptual understanding. This assumption is underlined by the fact that participants in the annotation condition reported higher self-assessed knowledge gain compared to participants in other conditions.

Moreover, we found that collaborative learners did not perform better in conceptual understanding than individual learners (H2c), which is in contrast to related research (Kirschner et al., 2011; Retnowati et al., 2016). Results on learning processes revealed that learning activity and cognitive load appeared to be relatively similar between individuals and dyads (H1b). Thus, considering previous work (cf. Janssen & Kirschner, 2020), we conclude that the used environment supported learning in such a way that not only individuals, but also collaborative learners could benefit.

In sum, by considering different antecedents of learning and learning processes, as suggested by situative approaches (Greeno & Engeström, 2014; Janssen & Kirschner, 2020), we were able to gain a deeper understanding of how (individual and collaborative) learners use enhanced tools to learn (cf. Fiorella & Mayer, 2016) and found that enhanced tools impact learners’ focus on learning in enhanced video-based environment, leading to different strategies to use these tools for learning, which results in different learning outcomes.

### Limitations and future work

This study has several limitations, which we will discuss here, along with recommendations for future research. First, to investigate our study goals, we conducted an experimental laboratory study in a co-located face-to-face context. Thus, high internal validity could be achieved because several disturbing variables could be excluded or controlled. However, especially regarding intrinsic and extrinsic motivation, learning in the laboratory cannot be compared with learning in everyday contexts (cf. motivational processes, Mayer, 2014). Hence, future research should conduct field studies as they allow for reasonable generalizations by implying high situational representativity and, thus, external validity (Rack & Christophersen, 2009). Second, the success of computer-supported (collaborative) learning depends on many different aspects (e.g., Janssen & Kirschner, 2020). Although we pursued a holistic approach by including different aspects that shape learning, many other variables play a decisive role: for example, student characteristics, such as the ability for self-regulated learning processes (Janssen & Kirschner, 2020). Future research should increasingly address such aspects and their mutual influence on learning processes and outcomes. Third, our results on learning outcomes (i.e., generative learning and conceptual understanding) suggest, on one hand, that investigating long-term effects of learning with enhanced video environments could provide new insights, which should be addressed in future work. On the other hand, further methods to measure quality of hypervideo products—for example by deeply considering text quality of annotation texts—are recommended for future research. Forth, concerning learning processes, we suggest addressing the close relationship between micro- and task-actions in future research, for example by conducting behavior sequence analyses (see for example, Sinha et al., 2014). Last, cognitive load should be considered by using measurements for intrinsic, extraneous, and germane load with validated instruments (e.g., Klepsch et al., 2017) and by focusing on collaborative load (e.g., Kontogiorgos & Gustafson, 2021).

## Conclusion

Enhanced video-based environments could provide answers to the question of how we can support *effective* learning. In the present work, we aimed to tackle the conflicting results on whether enhanced tools support generative learning and conceptual understanding in individuals and groups. The scientific relevance of this study lied on a holistic approach to investigate learning. We systematically and simultaneously investigated the effects of different antecedents of learning (different enhanced tools: annotations vs. hyperlinks; different settings: individual vs. collaborative learning) on learning processes (i.e., learning activity and cognitive load), learning outcomes (i.e., hypervideo product quality and conceptual understanding), and their relations. Our results suggest that hyperlinks are more suited to create own information structures of the learning material (i.e., hypervideo products) compared to annotations. Results further indicate that this depends on a frequent use of basic video control tools (such as play, pause, and rewind). In contrast, annotations foster self-assessed knowledge gain and help learners to deepen their conceptual understanding of the learning material—but only when they perceive the learning material not too difficult. Moreover, we found a marginal superiority of collaborative over individual learnings in constructing hypervideo products, indicating that generative learning might be fostered through collaboration. Our study sheds light into the different aspects that shape learning and highlights the importance to investigate them holistically.
